# One-minute stages are optimal for maximal exercise testing in endurance male and female athletes

**DOI:** 10.1016/j.clinsp.2025.100829

**Published:** 2025-11-21

**Authors:** Alice D. Campos, Claudio A.B. de Lira, Rodrigo L. Vancini, João V R de Freitas, Katja Weiss, Thomas Rosemann, Beat Knechtle, Marilia S. Andrade

**Affiliations:** a Department of Orthopedics and Traumatology, Universidade Federal de São Paulo, São Paulo, SP, Brazil; b Human and Exercise Physiology Division, Faculdade de Educação Física e Dança, Universidade Federal de Goiás, Goiânia, GO, Brazil; c ES Center for Physical Education and Sports, Universidade Federal do Espírito Santo, Vitória, ES, Brazil; d Institute of Primary Care, University Hospital of Zurich, Zurich, Switzerland; e Medbase St. Gallen Am Vadianplatz, St. Gallen, Switzerland; f Department of Physiology, Universidade Federal de São Paulo, São Paulo, SP, Brazil

**Keywords:** Maximal oxygen uptake, Incremental exercise test, Cycle ergometry, Athletes, Endurance performance, Sex differences, Ventilatory thresholds, Test efficiency

## Abstract

**Objective:**

Over the last 40 yr., there has been a relative consensus that incremental exercise tests used to determine Maximal Oxygen uptake (V̇O_2_max), maximal aerobic intensity, and ventilatory thresholds should last between 8 and 12 min. However, recent studies have questioned this recommendation, suggesting that it may be outdated, although this is not yet established in the literature. This study compared V̇O_2_max, Maximum Aerobic Power (MAP), and ventilatory thresholds between two incremental exercise protocols in amateur endurance athletes.

**Methods:**

Thirteen women and 15 men (aged 24–53 yr) completed two maximal progressive tests, performed in random order (short-term and long-term), with a 2- to 4-day interval. In the short-term test, the workload increased by 25 W per minute, while in the long-term test, the workload increased by 25 W every 3 min. Both protocols were performed until exhaustion. The variables assessed included ventilatory threshold (VT), Respiratory Compensation Point (RCP), V̇O_2_max, MAP, Maximal Heart Rate (HRmax), and maximal oxygen pulse.

**Results:**

No significant differences were found between the short-term (8–12 min) and long-term (19–28 min) protocols for V̇O_2_max (*p* = 0.79), HRmax (*p* = 0.715), or maximal oxygen pulse (*p* = 0.40). However, absolute and relative MAP (*p* < 0.001) and power at RCP (*p* < 0.001) were significantly lower in the short-term protocol. Moreover, men showed a greater reduction in MAP during long-term tests than women.

**Conclusions:**

These findings suggest that 1-min stage tests are more time-efficient and effective for assessing V̇O_2_max and MAP.

## Highlights

• V̇O_2_max is similar between 1- and 3-min stages for men and women.
• The test protocol did not affect %V̇O_2_max, HR or power at Ventilatory threshold.
• Female athletes show less MAP reduction in long tests than males.
• Relative MAP reductions are similar between sexes in both protocols.
• Protocols with 1-min stages are more suitable for evaluating V̇O_2_max and MAP.

## Introduction

Cardiopulmonary Exercise Testing (CPX) is a versatile and informative tool for assessing the functional capacity of patients and athletes. CPX allows for the real-time analysis of gas exchange (oxygen and carbon dioxide) and pulmonary ventilation during incremental exercise through breath-by-breath measurements.^1^ Among the many CPX variables, Maximal Oxygen consumption (V̇O_2_max) is a key indicator, reflecting the maximal capacity of the cardiopulmonary system.^2^ Other variables, such as maximal aerobic intensity, oxygen pulse, and heart rate, provide insights into training status, training effectiveness, and the magnitude of effort performed during testing. In addition to parameters measured during maximal exertion, the Ventilatory Threshold (VT) and Respiratory Compensation Point (RCP) are valuable markers. Ventilatory threshold is the point at which ventilation begins to increase disproportionately to V̇O_2_, while RCP is reached when ventilation exceeds the increase in V̇CO_2_.^1^

Nevertheless, the specific protocol adopted for conducting the test, such as the duration allocated to each stage of exercise intensity and the total length of the assessment, has the potential to substantially influence the physiological responses measured and, consequently, the results obtained.

There is a general consensus that incremental exercise tests should last between 8- and 12-min, based on findings from Buchfuhrer et al.^3^ However, this study, conducted with only 12 male participants, may not provide a comprehensive recommendation for all populations. Additionally, although not reported in the text, the participants exhibited a ventilatory threshold at only 50 % of V̇O_2_max, suggesting that their results may only be applicable to this type of population, which should not be athletes, once the percentage of V̇O_2_max at VT is lower than the expected for athletes.^2^ Another interesting point is that the study was conducted only with men, yet the results were generalized to women without confirming whether their cardiorespiratory responses follow the same patterns during an incremental exercise test. Since that study, conducted in 1983, some researchers have employed incremental protocols for V̇O_2_max determination lasting well beyond 12 min.^4^, ^5^, ^6^ Although there is some evidence that longer tests may result in lower V̇O_2_max values, this is still a matter of debate in the literature.^6^^,^^7^ Moreover, the effects of protocol on ventilatory threshold remain unclear.

Recent trends indicate increased female participation in sports. In 2018, women accounted for 50 % of participants in running events.^8^ The 2024 Paris Olympics were the first to ensure equal representation of male and female athletes.^9^ Despite the increasing participation of women in sports, the majority of research in Sports and Exercise Medicine (SEM) continues to focus on male participants.^10^ In 2014, approximately 65 % of participants in SEM studies were male, with females significantly underrepresented in leading SEM journals, including Medicine and Science in Sports and Exercise, British Journal of Sports Medicine, and the American Journal of Sports Medicine.^10^

The underrepresentation of female athletes in scientific research raises concerns, given that physiological and anatomical differences between the sexes impact athletic performance.^11^ These differences include variations in V̇O_2_max, fatigue mechanisms, substrate utilization, muscle tissue characteristics, and thermoregulation.^12^ Moreover, endurance training responses differ by sex. A recent study by the Molecular Transducers of Physical Activity Consortium reported that 58 % of training-regulated features exhibited sex-specific responses.^13^ Thus, applying findings from male populations to female athletes without scientific validation may lead to inaccurate conclusions.^14^

The present study aimed to compare V̇O_2_max, VT and RCP values between two incremental exercise test protocols (3-min per stage vs. 1-min per stage) in male and female amateur endurance athletes. The authors hypothesized that the longer protocol (3-min per stage) would negatively impact maximal aerobic power in both sexes due to the increased time above the VT. However, the authors anticipated that the impact would be smaller for women, as they typically have a higher percentage of oxidative muscle fibers than men.

## Material and methods

### Ethics statement

The Human Research Ethics Committee of the Federal University of São Paulo reviewed and approved all experimental procedures (protocol n° 0793/2023, approved on March 15, 2024). The study complied with the Declaration of Helsinki principles. After receiving information about the study’s objectives, procedures, risks, benefits, and privacy protections, all participants provided informed consent.

### Participants

The study included 28 participants ‒ 13 women and 15 men ‒ who were amateur endurance athletes (runners, cyclists, and triathletes) aged 24 to 53 years. Participants were recruited via sports coaching services and social media. Table 1 presents participants’ characteristics, including age, body height, body weight, and Body Mass Index (BMI).

**Table 1:** Characteristics of the participants.

	Women	Men	p-value	Effect size
**Age (years)**	33.3 ± 9.4	36.1 ± 8.9	0.420	0.30
**Body mass (kg)**	63.7 ± 5.2	77.1 ± 8.2	<0.001	1.95
**Height (m)**	1.65 ± 0.06	1.74 ± 0.08	0.002	1.27
**BMI (kg/m²)**	23.4 ± 2.5	25.5 ± 2.2	0.040	0.89

BMI, Body Mass Index. Data are expressed as mean ± standard deviation.

The inclusion criteria for participants were as follows: a) Aged 20–60 years and b) Engaged in endurance training for at least 2 years, with a minimum frequency of three sessions per week in running, cycling, or triathlon. All participants completed the Physical Activity Readiness Questionnaire (PAR-Q). Those under 49 years old who answered “no” to all questions were included. Participants aged 50-years or older, or those who answered “yes” to at least one PAR-Q question, were required to undergo medical clearance before participating.

The exclusion criteria included the following: a) Presence of comorbidities (e.g., orthopedic injuries, uncontrolled cardiovascular, respiratory, or metabolic diseases, or acute infections); b) Fewer than 2-years of sports practice; and c) Incomplete maximal test performance.

This study was conducted in accordance with the Strengthening the Reporting of Observational Studies in Epidemiology (STROBE) guidelines.

### Study design

Participants visited the Exercise Physiology Laboratory at UNIFESP twice. During these visits, they performed two maximal progressive exercise tests ‒ a short-term test and a long-term test) ‒ on separate days, with a 2- to 4-day interval between tests. The order of tests was randomized. Participants avoided stimulants (e.g., caffeine or tea) for at least 8 h before testing and were instructed to maintain regular hydration and wear appropriate athletic clothing.

### Physical evaluation

Body height (m) and total body mass (kg) were measured using a calibrated wall stadiometer and a scale (Filizola®, São Paulo, Brazil), respectively. Measurements were recorded to the nearest 0.1 cm and 0.1 kg. BMI was calculated by dividing the total body mass by height squared.

### Cardiopulmonary exercise test

Participants performed both the short-term and long-term exercise tests on a cycle ergometer (New Excalibur Sport, Lode, Nederland) to assess VT, RCP, V̇O_2_max, Maximum Aerobic Power (MAP), and maximal oxygen pulse. A metabolic system (Quark CPET, Cosmed®, Italy) equipped with a respiratory flow sensor and fast-response O_2_ and CO_2_ analyzers, thermally adjusted for barometric pressure, temperature, and humidity, was used to monitor pulmonary gas exchange. This method has been validated for accuracy and reliability in calculating V̇O_2_max.^15^ Respiratory and metabolic data were collected breath-by-breath and averaged every 20 s for analysis. Calibration was performed according to the manufacturer’s instructions before each test. Heart rate was monitored using a chest strap heart monitor (H9, Polar, Finland).

The evaluations began with a 1-min rest interval, which could be extended if participants showed signs of hyperventilation, indicating anxiety or discomfort. The test commenced with a 3-min warm-up at 50 W for both men and women. Immediately after the warm-up, the incremental phase began. In the short-term protocol, the workload increased by 25 W every 1 min.^16^^,^^17^ In the long-term protocol, the workload increased by 25 W every 3 min.^18^ Participants maintained a cadence of 70 rpm throughout the test and were instructed to continue until they reached voluntary exhaustion. Tests were terminated if cadence dropped below 60 rpm despite verbal encouragement from the examiner.^19^ All tests were administered by the same examiner experienced in conducting graded exercise testing.

The Borg scale was used to assess perceived exertion at the end of each stage.^20^ Cardiovascular, metabolic, and respiratory variables were monitored for 2 min after the test. Using graphical methods, the VT, RCP, and V̇O_2_max were determined.^21^ VT was defined as the point where a break in the linearity of V̇E/V̇O_2_ occurred, along with an increase in end-tidal O_2_ pressure (PETO_2_). The respiratory compensation point was identified by a disruption in the linearity of V̇E/V̇CO_2_ and a decline in end-tidal CO_2_ pressure (PETCO_2_). Maximal oxygen uptake was determined by the presence of a plateau in V̇O_2_ curve (increase < 150 mL.min^-1^) despite increasing workload.^22^ In cases where no V̇O_2_ plateau was observed, the test was considered maximal if the respiratory quotient (V̇CO_2_/V̇O_2_) exceeded 1.1, the maximum predicted heart rate was achieved, and perceived exertion reached 20 on the Borg scale.^20^

The oxygen pulse was calculated as the ratio of V̇O_2_ (mL/min) to heart rate (bpm). The time intervals between VT and RCP and between RCP and the end of the test were also analyzed.

Maximal Aerobic Power (MAP) was defined as the maximal load achieved during the test. The power at each threshold was recorded as VT power and RCP power. These power values were reported in both absolute terms and relative to body mass.

### Statistical analysis

Descriptive data were presented as mean ± standard deviation. The Shapiro-Wilk test confirmed the normality of the data, and the Levene test indicated homogeneous variances. The sample size was calculated based on an effect size of 0.30 (20), a significance level of 0.05, and a power of 0.80, resulting in a required sample of 24 participants.

Differences in anthropometric data between sexes were evaluated using an independent t-test. A two-way repeated measures analysis of variance was performed to assess the effects of the test protocol, sex, and their interaction (test protocol × sex). When needed, Sidak’s post hoc test was used. Statistical significance was set at *p* < 0.05. All analyses were conducted using SPSS version 26.0 (SPSS, Inc., USA).

## Results

There was no significant difference in age between the sexes (*p* = 0.422). However, body mass, height, and BMI were higher in male participants (*p* < 0.01, *p* = 0.002, and *p* = 0.04, respectively; Table 1). As expected, the long-term test lasted significantly longer than the short-term test for both sexes (*p* < 0.001). The short-term test lasted approximately 8 and 12 min for the female and male groups, respectively, while the long-term test lasted approximately 19 and 28 min (Table 2).

**Table 2:** Values reached at ventilatory threshold during the short and long test by female (*n* = 13) and male (*n* = 15) athletes.

	Sex	Short test	Long test	Mean difference (95 % CI)	ANOVA	*F*	p-value	Effect size
**VT values**								
V̇O_2_ at VT ( %)	F	63.3 ± 6.1	64.7 ± 7.9	1.1 (−3.9 to 6.2)	Test effect	3.637	0.068	0.123
	M	61.1 ± 9.2	66.4 ± 11.7	5.3 (0.5 to 10.1)	Sex effect	0.270	0.870	0.001
					Interaction effect	1.521	0.228	0.055
Power at VT (W)	F	121.2 ± 30.4^a^	121.2 ± 22.5^a^	0.0 (−8.7 to 8.7)	Test effect	0.205	0.655	0.008
	M	178.3 ± 48.1	175.0 ± 41.2	3.3 (−9.3 to 16.0)	Sex effect	16.34	<0.001	0.386
					Interaction effect	0.205	0.655	0.008
Power at VT (W/kg)	F	1.90 ± 0.48	1.90 ± 0.33	0.003 (−0.14 to 0.15)	Test effect	0.163	0.690	0.006
	M	2.32 ± 0.59	2.28 ± 0.57	0.036 (−0.101 to 0.174)	Sex effect	4.400	0.046	0.145
					Interaction effect	0.111	0.742	0.004
HR (bpm)	F	140.7 ± 18.6	142.1 ± 16.1	−1.4 (−9.9 to 7.2)	Test effect	1.527	0.228	0.055
	M	137.9 ± 13.4	142.4 ± 13.0	−4.4 (−10.5 to 1.5)	Sex effect	0.053	0.820	0.002
					Interaction effect	0.424	0.521	0.016
O_2_Pulse (mL/beats)	F	12.1 ± 2.4^a^	12. ± 2.3^a^	−0.03 (−0.85 to 0.78)	Test effect	3.610	0.068	0.122
	M	17.2 ± 5.1	18.6 ± 4.6	−1.4 (−2.6 to −0.1)	Sex effect	16.300	<0.001	0.385
					Interaction effect	3.263	0.082	0.111
Time VT – RCP (min)	F	2.1 ± 0.4^a^^,^^b^	4.8 ± 1.2^a^	−2.6 (−3.3 to −2.0)	Test effect	68.350	<0.001	0.724
	M	2.8 ± 0.8^b^	6.6 ± 2.8	3.7 (−5.1 to −2.2)	Sex effect	6.109	0.020	0.190
					Interaction effect	1.833	0.187	0.066

a p < 0.05 (different from the other sex for the same test).

b p < 0.05 (different from the other test for the same sex).

### Ventilatory threshold

Regarding the VT, the test protocol (short-term vs. long-term) did not significantly affect %V̇O_2_max (*p* = 0.068), HR (*p* = 0.228), oxygen pulse (*p* = 0.068), or power at VT (*p* = 0.655). However, male participants had higher oxygen pulse (*p* = 0.003 and *p* < 0.001, respectively) and power (*p* = 0.001 and *p* < 0.001, respectively) than female participants during both protocols. Additionally, males spent more time between VT and RCP than females (*p* = 0.009 for short-term and *p* = 0.050 for long-term; Table 2).

### Respiratory compensation point

No significant effect of the protocol or sex was observed on V̇O_2_max, HR, or oxygen pulse at RCP. However, the power reached RCP was lower in the long-term test than in the short-term test for both sexes (*p* = 0.001 for males and *p* = 0.050 for females). Time spent above RCP was longer in the long-term test of both groups (*p* < 0.001; Table 3). Male athletes also achieved significantly higher oxygen pulse and power at RCP than females in both protocols (Table 3).

**Table 3:** Values reached at the respiratory compensation point during the short and long test by female (*n* = 13) and male (*n* = 15) athletes.

	Sex	Short test	Long test	Mean difference (95 % CI)	ANOVA	F	p-value	Effect size
**RCP values**								
V̇O_2_ at RCP ( %)	F	81.3 ± 6.2	80.7 ± 9.2	0.5 (−5.0 to 6.0)	Test effect	0.319	0.577	0.012
	M	79.4 ± 8.2	82.0 ± 7.0	−2.1 (−7.7 to 2.5)	Sex effect	0.017	0.896	0.001
					Interaction effect	0.732	0.400	0.027
Power at RCP (W)	F	173.1 ± 33.0^a^^,^^b^	163.5 ± 26.2^a^	9.6 (−2.0 to 21.2)	Test effect	16.125	<0.001	0.383
	M	248.3 ± 47.7^b^	231.7 ± 44.8	16.7 (8.1 to 25.2)	Sex effect	24.079	<0.001	0.481
					Interaction effect	1.161	0.291	0.043
Power at RCP (W/kg)	F	2.72 ± 0.53	2.57 ± 0.42	0.152 (0.006 to 0.298)	Test effect	14.90	0.001	0.364
	M	3.25 ± 0.69	3.03 ± 0.64	0.223 (0.087 to 0.359)	Sex effect	5.05	0.033	0.163
					Interaction effect	0.533	0.472	0.020
HR (bpm)	F	157.4 ± 15.2	160.2 ± 14.5	−2.7 (−9.6 to 4.1)	Test effect	2.849	0.103	0.099
	M	159.9 ± 12.2	163.1 ± 12.8	−3.2 (−7.1 to 1.8)	Sex effect	0.324	0.574	0.012
					Interaction effect	0.015	0.904	0.001
O_2_Pulse (mL/beats)	F	13.8 ± 2.7^a^	13.3 ± 2.1^a^	0.49 (−0.57 to 1.55)	Test effect	0.043	0.838	0.002
	M	19.5 ± 5.3	19.8 ± 4.3	−0.3 (−1.6 to 0.9)	Sex effect	17.685	<0.001	0.405
					Interaction effect	1.111	0.302	0.041
Time RCP – Max (min)	F	2.5 ± 0.6^b^	5.9 ± 1.7	−3.3 (−4.2 to −2.4)	Test effect	157.99	<0.001	0.859
	M	3.3 ± 0.5^b^	6.5 ± 1.6	−3.2 (−3.9 to −2.5)	Sex effect	2.855	0.103	0.099
					Interaction effect	0.050	0.826	0.002

a p < 0.05 (different from the other sex for the same test).

b p < 0.05 (different from the other test for the same sex).

### Maximal values

V̇O_2_, HR, and oxygen pulse did not differ between protocols at maximal exercise intensity, but MAP was lower in the long-term test than in the short-term test (*p* < 0.001 for both sexes). Interestingly, the results showed an interaction effect for MAP, indicating that the magnitude of the test’s effect on MAP differed between sexes. Men exhibited a greater reduction in MAP during the long-term test than women (Table 4).

**Table 4:** Maximal values reached during the short and long test by female (*n* = 13) and male (*n* = 15) athletes.

	Sex	Short test	Long test	Mean difference (95 % CI)	ANOVA	F	p-value	Effect size
**Maximal values**								
V̇O_2_ max (mL/kg/min)	F	42.2 ± 7.7^a^	41.9 ± 7.4^a^	0.2 (−2.5 to 3.0)	Test effect	0.072	0.791	0.003
	M	50.1 ± 10.4	50.8 ± 7.5	−0.8 (−3.5 to 1.9)	Sex effect	7.406	0.011	0.222
					Interaction effect	0.263	0.612	0.010
MAP (W)	F	238.5 ± 44.0^a^^,^^b^	203.8 ± 35.1^a^	34.6 (24.8 to 44.4)	Test effect	215.46	<0.001	0.892
	M	333.3 ± 54.8^b^	281.7 ± 49.5	51.7 (43.4 to 59.9)	Sex effect	24.24	<0.001	0.483
					Interaction effect	8.415	0.007	0.245
MAP (W/kg)	F	3.7 ± 0.7	3.2 ± 0.6	0.543 (0.405 to 0.681)	Test effect	177.6	<0.001	0.872
	M	4.3 ± 0.8	3.6 ± 0.5	0.681 (0.553 to 810)	Sex effect	4.03	0.055	0.134
					Interaction effect	2.26	0.144	0.080
HR (bpm)	F	174.7 ± 12.2	175.1 ± 10.6	−0.3 (−3.9 to 3.1)	Test effect	0.136	0.715	0.005
	M	180.0 ± 10.2	178.8 ± 13.4	1.2 (−2.0 to 4.4)	Sex effect	1.110	0.302	0.041
					Interaction effect	0.513	0.480	0.019
O_2_Pulse (mL/beats)	F	15.3 ± 2.5^a^	15.3 ± 2.5^a^	0.0 (−0.9 to 0.9)	Test effect	0.729	0.401	0.027
	M	21.3 ± 4.5	21.9 ± 4.1	−0.6 (−1.8 to 0.6)	Sex effect	23.529	<0.001	0.475
					Interaction effect	0.711	0.407	0.027
Time (m)	F	8.48 ± 1.7^a^^,.^^b^	18.9 ± 4.05^a^	10.4 (8.5 to 12.3)	Test effect	427.68	<0.001	0.943
	M	12.2 ± 2.1^b^	28.1 ± 6.1	15.8 (14.1 to 17.6)	Sex effect	22.73	<0.001	0.466
					Interaction effect	18.30	<0.001	0.413

a p < 0.05 (different from the other sex for the same test).

b p < 0.05 (different from the other test for the same sex).

Regarding sex differences after the short- and long-term tests, female athletes demonstrated significantly lower values than male athletes for V̇O_2_max (*p* = 0.034 and *p* = 0.004, respectively), O_2_ pulse (*p* < 0.001 for both tests), and MAP (*p* < 0.001 for both tests; Table 4).

## Discussion

This study investigated the effects of sex (male or female) and incremental test protocols (short-term vs. long-term) on VT, RCP, and maximal values. The key findings were as follows: a) Maximal values for V̇O_2_, HR, and O_2_ pulse were not significantly different between the two protocols for both sexes; b) Absolute and body mass-relative MAP values were higher in the short-term protocol than in the long-term protocol for both sexes; c) No significant differences were observed between the short- and long-term protocols for variables assessed at VT; and d) Absolute and body mass-relative power at RCP were lower in the long-term protocol than in the short-term protocol for both sexes.

Maximal V̇O_2_, HR, and O_2_ pulse did not differ significantly between the short-term (8 and 12 min for female and male groups, respectively) and long-term (19 and 28 min for female and male groups, respectively). These findings challenge the traditional recommendation that an 8- to 12-min incremental test protocol is required to elicit valid VO_2_max values, as initially proposed by Buchfuher et al.^3^ based on a small sample size (*n* = 12) of male participants. Exercise testing guidelines from the American College of Sports Medicine also endorse this protocol length.^23^ Accordingly, the present findings advance the understanding of incremental exercise testing by demonstrating that valid V̇O_2_max measurements can be obtained with protocols exceeding the traditionally recommended duration. Nevertheless, and considering the absence of differences in V̇O_2_max between protocols, the shorter protocol appears to be the most appropriate option because it is less time-consuming.

Many other experimental studies have involved repeat determinations of V̇O_2_max using incremental exercise tests. One of these studies analyzed the effect of stage duration on V̇O_2_max, maximal heart rate, and maximal running speed among male runners by comparing three incremental running tests with stage durations of 1, 3, and 6 min.^24^ Another study evaluated male runners who performed maximal incremental tests with stage durations of 1, 2, and 3 min. In these tests, V̇O_2_max and maximum running speed were analyzed.^25^ Both studies observed that V̇O_2_max values were similar between tests, whereas the maximal running speed was higher with shorter stages.

Regarding tests performed on a cycle ergometer, similar findings were observed among triathletes.^26^ The authors compared a short protocol, where the athletes increased power output every 1 min (short protocol), with a protocol requiring increases every 3 min (long protocol). Similarly, a higher maximal power output was identified in the short protocol, while similar values for the V̇O_2_ peak were observed in both protocols. A review study comparing V̇O_2_max values from cycle ergometer tests lasting from 5 to 28 min concluded that these tests should last between 7 and 26 min to elicit valid V̇O_2_max values; however, no maximal power data were studied.^27^ Therefore, our results are in line with the literature.

In terms of absolute values for MAP, a significant effect of the test was observed. Specifically, the long-term test resulted in lower MAP values than the short-term test for both sexes. Additionally, a significant interaction effect was identified, indicating that sex influences the MAP differently between protocols. Male athletes exhibited a greater reduction in absolute MAP values in the long-term test than female athletes. The higher MAP observed in protocols with shorter stage durations may be attributed to greater anaerobic contributions during the later stages, resulting from delayed buildup and increased core temperature ‒ factors that contribute to local muscle fatigue.^28^ Furthermore, correlations between the maximum work rate and the duration of Graded Exercise Tests (GXTs) indicate that shorter test durations result in increased work rates relative to time (W.s^-^¹).^29^

For MAP relative to body mass (W/kg), the long-term test also produced lower values than the short-term test. However, the magnitude of the reduction was similar for both sexes. No significant difference was found between the sexes in relative MAP values. Previous literature similarly indicates that longer test protocols result in lower MAP values than shorter protocols.^26^ It is noteworthy that the effect of sex on MAP was only observed in absolute values, not in values normalized to body mass. This finding could be attributed to the fact that although men demonstrated higher absolute MAP values, the relative MAP values for the longest test were similar between the sexes due to higher body mass for men.

Values for %V̇O_2_max, power, HR, and O_2_ pulse measured at the VT were similar between the short- and long-term protocols. During moderate exercise below the VT, ATP production is primarily achieved through oxidative phosphorylation and type I muscle fiber recruitment.^30^ Therefore, lactate concentration remains low and stable, without any significant accumulation of lactate or *H*^+^ ions in the blood.^31^ Consequently, it was not expected that the long-term protocol would alter VT values, as additional time spent below the VT does not disrupt homeostasis.

For the RCP, the %V̇O_2_max, HR, and O_2_ pulse were also similar between protocols, although absolute and relative power values were lower in the long-term test than in the short-term test. This outcome may be related to the longer time spent between the VT and RCP in the long-term protocol. Between the VT and RCP, an isocapnic buffering phase occurs, during which bicarbonate buffers the increased lactate and *H*^+^ ions.^32^ Spending more time in this phase could account for the reduced power output associated with the RCP.

In the present study, it was shown that HRmax is independent of sex (*p* = 0.30). As expected, men demonstrated a V̇O_2_max that was 18 % higher than that of women (*p* = 0.01).^33^ An evaluation of 37 male runners and 38 female runners with varying performance levels found that women’s V̇O_2_max values were 21 % lower than men’s.^33^ Maximal oxygen uptake is a key determinant of performance in endurance sports. Among elite marathon runners, the performance gap between men and women ranges from 10 % to 12 %.^34^ Despite increased female participation and notable performances in competitions, at the Paris 2024 Olympics, the best female marathon time (2:22:55)^35^ would rank 68th among male competitors.

Extensively studied are the physiological differences between men and women that contribute to men’s superior endurance performance. Men generally have a larger left ventricular mass, resulting in a greater stroke volume and oxygen pulse. Additionally, men have a higher blood volume and hemoglobin concentration (12 %–15 %).^36^ Similar to their cardiac structure, women have smaller lungs and narrower airways.^37^ Together, these physiological differences create greater central limitations to whole-body exercise performance in females.

In practical terms, the findings of the present study suggest that incremental treadmill protocols with 1-minute stage durations may represent a more efficient alternative to 3-minute stages because it is less time-consuming. Such protocols require less time to administer while providing comparable outcomes for both maximal and threshold variables in male and female athletes alike.

### Study limitations

The absence of a significant difference in V̇O_2_max (mL/kg/min) between the two tests may have been influenced by the small sample size, thereby increasing the likelihood of a Type II error. Consequently, future studies employing larger sample sizes are recommended to confirm the similarity between protocols. Furthermore, it's crucial to recognize that the findings of this study are only applicable to the examined sample of amateur athletes and should not be extrapolated to other populations.

## Conclusion

Although an incremental test with 3-min stages produces similar V̇O_2_max, HRmax, and O_2_Pulsemax values as a test with 1-min stages for both sexes, absolute and relative PAM values, as well as power at the RCP, are also lower for both sexes. Therefore, protocols with 1-min stages are more suitable for evaluating V̇O_2_max and MAP because it is less time-consuming. Therefore, protocols with 1-min stages should be considered for cardiorespiratory evaluation of amateur female and male athletes. Additionally, the effect of the test on absolute PAM is greater for male athletes than for female athletes, but power values relative to body mass do not differ significantly between sexes. Finally, shorter protocols are preferable in sports clinics for time-saving and accurate VO₂max assessment for male and female athletes.

## Funding

This research did not receive any specific grant from funding agencies in the public, commercial, or not-for-profit sectors.

## Data availability statement

The datasets generated and/or analyzed during the current study are available from the corresponding author upon reasonable request.

## Declaration of competing interest

The authors declare no conflicts of interest.
